# Serosurveillance for pandemic influenza A (H1N1) 2009 virus infection in domestic elephants, Thailand

**DOI:** 10.1371/journal.pone.0186962

**Published:** 2017-10-26

**Authors:** Weena Paungpin, Witthawat Wiriyarat, Kridsada Chaichoun, Ekasit Tiyanun, Nareerat Sangkachai, Don Changsom, Kanaporn Poltep, Parntep Ratanakorn, Pilaipan Puthavathana

**Affiliations:** 1 Department of Microbiology, Faculty of Medicine Siriraj Hospital, Mahidol University, Bangkok, Thailand; 2 The Monitoring and Surveillance Center for Zoonotic Diseases in Wildlife and Exotic Animals, Faculty of Veterinary Science, Mahidol University, Nakhon Pathom, Thailand; 3 One Health Animal Clinic, Mahidol University Nakhon Sawan Campus, Nakhon Sawan, Thailand; 4 Center for Research and Innovation, Faculty of Medical Technology, Mahidol University, Nakhon Pathom, Thailand; 5 Center for Emerging and Neglected Infectious Disease, Mahidol University, Nakhon Pathom, Thailand; Icahn School of Medicine at Mount Sinai, UNITED STATES

## Abstract

The present study conducted serosurveillance for the presence of antibody to pandemic influenza A (H1N1) 2009 virus (H1N1pdm virus) in archival serum samples collected between 2009 and 2013 from 317 domestic elephants living in 19 provinces situated in various parts of Thailand.

To obtain the most accurate data, hemagglutination-inhibition (HI) assay was employed as the screening test; and sera with HI antibody titers ≥20 were further confirmed by other methods, including cytopathic effect/hemagglutination based-microneutralization (microNT) and Western blot (WB) assays using H1N1pdm matrix 1 (M1) or hemagglutinin (HA) recombinant protein as the test antigen. Conclusively, the appropriate assays using HI in conjunction with WB assays for HA antibody revealed an overall seropositive rate of 8.5% (27 of 317). The prevalence of antibody to H1N1pdm virus was 2% (4/172) in 2009, 32% (17/53) in 2010, 9% (2/22) in 2011, 12% (1/8) in 2012, and 5% (3/62) in 2013. Notably, these positive serum samples were collected from elephants living in 7 tourist provinces of Thailand. The highest seropositive rate was obtained from elephants in Phuket, a popular tourist beach city. Young elephants had higher seropositive rate than older elephants.

The source of H1N1pdm viral infection in these elephants was not explored, but most likely came from close contact with the infected mahouts or from the infected tourists who engaged in activities such as elephant riding and feeding. Nevertheless, it could not be excluded that elephant-to-elephant transmission did occur.

## Introduction

The outbreak of the 2009 pandemic influenza was initially reported in the Mexican town of La Gloria, Veracruz, in mid-February of 2009 [1]. Subsequently, the disease spread and led to the announcement of the pandemic phase by the World Health Organization on 11^th^ June 2009. This pandemic influenza A (H1N1) 2009 virus (H1N1pdm virus) is shown to be a quadruple reassortant whose genome was derived from 4 origins: the hemagglutinin (HA), nucleoprotein (NP) and nonstructural (NS) genomic segments from classical swine virus; the neuraminidase (NA) and matrix (M) segments from Eurasian avian-like swine virus; the polymerase basic protein 2 (PB2) and polymerase acidic protein (PA) segments from North American avian virus; and the polymerase basic protein 1 (PB1) segment from human H3N2 virus [2]. This was the evidence for interspecies transmission of influenza A viruses between different animal species or between humans and animals. Various subtypes of influenza A viruses had been found to infect several mammalian species [3]. Epidemiological study and molecular characterization suggested that the pandemic influenza viruses originated mostly from animals, in particular, pigs and birds [4].

Contact transmission of influenza viruses from humans to animals was frequent. Farmers frequently transmitted human influenza viruses to pigs. Humans are the major sources of novel influenza virus infection in domestic and captive animals. The H1N1pdm virus has been detected repeatedly in commercial pig farms worldwide [5–7]. Transmission of H1N1pdm virus from humans to animals had been reported in pigs, turkeys, skunks, cats, American badger, Bornean binturong, black-footed ferret, cheetahs, guinea pigs, dogs, giant panda and pet ferret [5, 8–16]. An interesting report was the H1N1pdm infection in free-ranging northern elephant seals living off the central coast of California [17]. H1N1pdm virus infection in many animal species, in particular the domestic animals that live closely with humans, is still largely unknown. The infections in various host species might favor the viral genetic changes (evolution, adaptation and gene reassortment) and increase risk of influenza pandemic.

Domestic elephants in Thailand and other Southeast Asian countries live in close contact with mahouts [18, 19]. As of 26 June 2017, the Thai Elephant Conservation Center reported the presence of about 2,700 domestic elephants (*Elephas maximus*) in Thailand [20]. Approximately 95% of them belong to private owners, and probably more than half of them work in tourism. Domestic elephants are at risk of getting infections from mahouts and tourists through close contact. Elephants have long life-spans of up to 70 years [21], and must frequently be exposed to influenza viruses, but it is not known whether they are susceptible to the infection.

Most studies have determined the prevalence of influenza virus infection in humans and various animal species by antibody detection using hemagglutination-inhibition (HI) assay. The standard protocol includes the step of removal of non-specific serum inhibitors with receptor- destroying enzyme (RDE) [22]. Incomplete removal of non-specific serum inhibitors will cause false positive results. Unfortunately, the protocol has no step to check that these serum inhibitors have been completely removed. It is also doubtful whether the same method of treatment could be applied to sera of all species. This study was the first to determine the prevalence of influenza virus infection in elephants. Serosurveillance for H1N1pdm antibody was conducted in domestic elephants using archival serum samples collected between 2009 and 2013. To obtain the accurate data, HI assay was used as the screening test; while microneutralization (microNT) assay for neutralizing antibody and Western blot (WB) assay for antibody to recombinant matrix 1 (rM1) or recombinant hemagglutinin (rHA) protein were verified for using as the confirmatory test. Due to the lack of anti-elephant Ig conjugate as the secondary antibody, this study verified the use of horseradish peroxidase-conjugated protein A/G (protein A/G-HRP) for serving as the alternative secondary detector in WB assay.

## Materials and methods

### Ethical issues

Use of elephant sera in this study was approved by the Ethics and Animal Care and Use Committee of the Faculty of Veterinary Science, Mahidol University (Permit Number: MUVS-2016-09-39). Human serum samples were the archived leftover samples from blood donors, an H1N1pdm patient and healthy volunteers. The blood donors and patient signed in the consent forms for using their sera on research purposes. Pre-pandemic sera were obtained from the healthy volunteers with verbal consent. Use of human sera in this study was approved by the Institutional Review Board of the Faculty of Medicine Siriraj Hospital, Mahidol University.

### The study virus

The pandemic influenza virus used in this study, A/Thailand/104/2009(H1N1), was propagated in Madin-Darby canine kidney (MDCK) cells (obtained from the American Type Culture Collection; CCL-34) maintained in viral growth media containing Eagle’s minimum essential medium (EMEM) (Gibco, Life Technologies, Grand Island, NY) supplemented with 2 μg/ml of trypsin-tosyl phenylalanyl chloromethyl ketone (trypsin-TPCK) (Sigma-Aldrich, St. Louis, MO). The full genomic sequence of this virus can be retrieved from GenBank. Its HA nucleotide sequence (accession number GQ169382) was 99.6% and the HA amino acid sequence (accession number ACR23302) was 99.4% identical to that of A/California/7/2009 pandemic virus (accession numbers FJ966974 for nucleotide sequence and AMV49023 for amino acid sequence).

### Archival elephant sera

A total of 317 archival elephant sera used in this study were kindly provided by the project on animal DNA fingerprint under the cooperation between the Asian Elephant Foundation of Thailand and the Monitoring and Surveillance Center for Zoonotic Diseases in Wildlife and Exotic Animals, Faculty of Veterinary Science, Mahidol University. These serum samples were collected between 2009 and 2013 from 291 elephants living in 19 provinces throughout Thailand and 26 elephants with unknown location. History of respiratory disease or any other clinical symptoms at time of blood collection in these elephants was not obtained.

### HI assay

The protocol for HI assay was modified after that previously described [23–25], such that one more step for removal of non-specific inhibitors using kaolin was added. Briefly, 30 μl of the test serum were mixed with 90 μl of RDE (Denka Seiken, Tokyo, Japan) and incubated overnight in a water bath at 37°C. The RDE-treated serum was added with 90 μl of 12.5% (v/v) kaolin in normal saline solution (NSS), incubated for 1 hour at room temperature, heat-inactivated at 56°C for 30 minutes, followed by adding with 60 μl of NSS. The serum tube was centrifuged at 3,000 x g for 10 minutes, and the supernatant collected was further treated for removal of non-specific agglutinator by absorbing with 30 μl of 50% goose red blood cells (RBC) for 1 hour at 4°C before centrifuging again. The supernatant was collected and the treated serum at the dilution of 1:10 was obtained.

TH104 pandemic virus was titrated by hemagglutination assay, and used as the test antigen at a concentration of 4 hemagglutination (HA) units/25 μl. In HI assay, a 25 μl volume of the treated serum was serially 2-fold diluted in phosphate buffer saline (PBS) pH 7.2 to obtain the initial serum dilution of 1:20, and added with 4 HA units of the test virus into V-bottom shaped microtiter plates in duplicate. The reaction plates were incubated at room temperature for 30 minutes before adding 50 μl of 0.5% goose RBC suspension, and further incubated for 30 minutes at 4°C before the HI antibody titer was determined. The reciprocal of the highest serum dilution that completely inhibited the hemagglutination reaction was assigned as the HI antibody titer. The positive serum control, treated serum control, RBC control and back titration of the test virus were included in each run. For calculation of geometric mean titer (GMT), the titer <20 was assigned as 10.

### MicroNT assay

The leftover quantities of treated serum from the HI assay were determined for neutralizing (NT) antibody using microNT assay which is based on appearance of the cytopathic effect (CPE) of the infected cell monolayers in adjunct with the hemagglutination assay of the culture supernatant (CPE/HA-based microNT assay). The test sera were serially 2-fold diluted and assayed from the initial dilution of 1:20 to 1:2,560 in EMEM (Gibco) in 96-well microculture plates in duplicate. A 60 μl volume of the diluted serum was mixed with 60 μl of the test virus (working concentration of 200 tissue culture infective dose 50 (TCID_50_)/100 μl); and the test virus at final concentration of 100 TCID_50_/well was obtained. The reaction plates were incubated at 37°C for 2 hours. A 100 μl volume of the serum-virus mixture was transferred onto MDCK monolayer maintained in EMEM supplemented with trypsin-TPCK; and then further incubated at 37°C for 2 days. The cell monolayers were examined for appearance of CPE; while the culture supernatants were determined for non-neutralized viruses by hemagglutination assay in which a 50 μl volume of the culture supernatant was mixed with 50 μl of 0.5% goose RBC suspension and incubated at 4°C for 30 minutes. The NT antibody titer was defined as the reciprocal of the highest serum dilution that inhibited the degree of CPE by 50% and showed ≤ 2+ degree of hemagglutination. The positive serum control, cell control, and the virus back titration were included in each run. GraphPad Prism6 was used to generate graph for displaying HI and NT antibody titers to H1N1pdm virus in elephant sera.

### WB assay for verifying the binding efficiency of protein A/G-HRP to elephant IgG

WB assay was performed to determine the binding efficiency of protein A/G-HRP (Thermoscientific, Rockford, IL) to elephant serum immunoglobulin (Ig) under both non-reducing and reducing conditions. Under the non-reducing condition, the binding efficiency of protein A/G-HRP to native form of elephant Ig could be determined; while under the reducing condition, the binding efficiency of protein A/G-HRP to heavy (H) and/or light (L) chain of Ig molecule could be demonstrated. Under the non-reducing condition, elephant serum was serially 2-fold diluted from the starting dilution of 1:100 to 1:800. The diluted serum was mixed with 4× non-reducing sample buffer (250 mM Tris Cl pH 6.8, 0.4% bromophenol blue, 40% glycerol) and loaded in electrophoresing gel (8% denaturing discontinuous sodium dodecyl sulphate-polyacrylamide gel electrophoresis-SDS-PAGE). Under the reducing condition, elephant serum at dilution of 1:50 was mixed with 4× reducing sample buffer (8% SDS, 250mM Tris Cl pH 6.8, 8% β-mercaptoethanol, 0.4% bromophenol blue, 40% glycerol) and boiled for 10 minutes prior to electrophoresing in 12% SDS-PAGE. The electrophoresed proteins in gel were then blotted onto a nitrocellulose membrane (Pall, Port Washington, NY) using Trans-Blot semidry transfer cell (Bio-Rad, Hercules, CA). The blotted membrane was blocked with 1% bovine serum albumin in PBS plus 0.1% Tween-20, followed by overnight incubation at 4°C with HRP conjugated-protein A/G as the detector. The solution mixture containing 3,3'-diaminobenzidine (Sigma-Aldrich, St. Louis, MO), 8% NiCl_2_ and 6% H_2_O_2_ was used as the chromogenic substrate. Human sera were included as the control to verify the molecular weight (MW) of Ig molecule using protein A/G conjugated with HRP or goat anti-human IgG, IgM, IgA (H+L) conjugated with HRP as the detector.

### WB assay for detection of antibody to H1N1pdm HA or M1 protein in elephant sera

The rHA protein kindly provided by BEI Resources through the NIH Biodefense and Emerging Infections Research Resources Repository, NIAID, NIH (catalog number NR-13691), and the rM1 protein purchased from eEnzyme®, Gaithersburg, MD (catalog number IA-M1-023P) were used as the test antigens in the WB assay. Both rHA and rM1 proteins were derived from A/California/04/2009 (H1N1) virus. The rHA protein was expressed in the baculovirus-insect cell system, while the rM1 protein was expressed in the *Escherichia coli* system. The test antigen was mixed with 4× reducing sample buffer as mentioned above and boiled for 10 minutes prior to electrophoresing in 12% SDS-PAGE. For antibody detection by WB assay, the test elephant sera were diluted to the dilution of 1:50, and protein A/G conjugate was used as the detector. In parallel, convalescent serum of an H1N1pdm patient was included as the positive control in each run.

## Results

### HI and microNT assays for antibody to H1N1pdm virus

**Fig 1:**
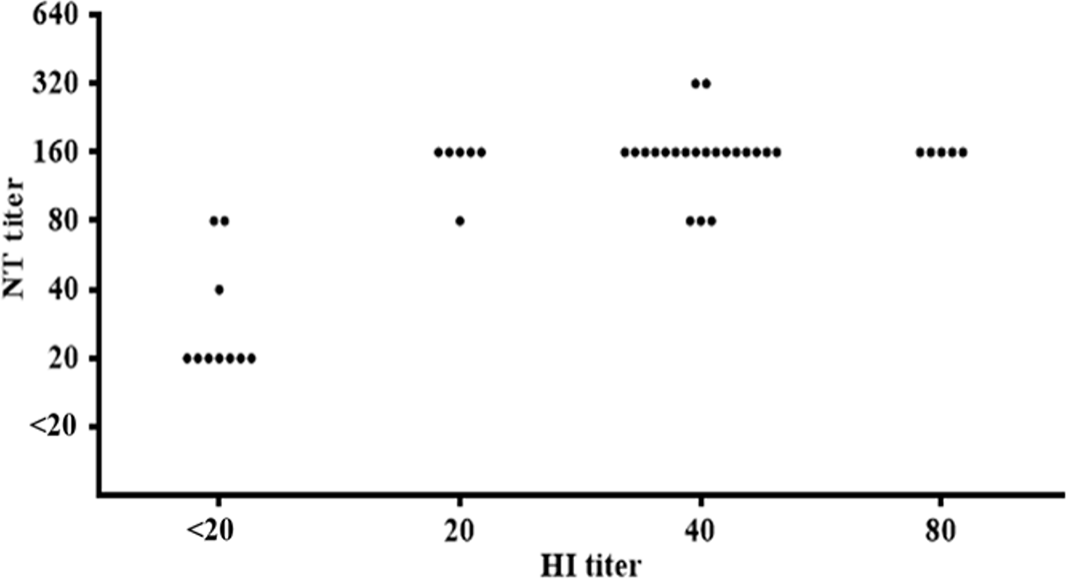
The correlation between HI and NT antibody titers to H1N1pdm virus in elephant sera. The figure displays 10 serum samples with HI titer <20, and 32 samples with HI titer ≥20.

A total of 317 elephant serum samples were screened for presence of antibody to H1N1pdm virus at the initial dilution of 1:20 by HI assay. There were 32 (10.1%) elephants that contained HI antibody titers in the range of 20–80 (Table 1), which led to the overall GMT of 11.5 (Table 1). These 32 HI antibody positive sera were further investigated by CPE/HA-based microNT assay; the result demonstrated that all of them had NT antibody titers in the range of 80–320 (GMT of 153.2) (Fig 1). Interestingly, all 10 HI negative control sera were also positive in microNT assay with the NT titers in the range of 20–80 (GMT of 28.3) (Fig 1). The data suggested that the NT antibody detected might be the result of H1N1pdm virus infection, the cross-reactive antibody induced by the other influenza virus strains, or a false positive result due to the neutralizing activity of serum inhibitor(s).

**Table 1:** Screening for antibody to H1N1pdm virus in elephant sera by HI assay.

Year	N	Number of serum samples at HI titers of					Number of serum samples with HI titers of ≥20 (%)
		<20	20	40	80	≥160	
2009	172	168	-	3	1	-	4 (2.3)
Jan-Jun	68	67	-	-	1	-	1 (1.5)
Jul-Dec	104	101	-	3	-	-	3 (2.9)
2010	53	34	3	12	4	-	19 (35.8)
2011	22	20	-	2	-	-	2 (9.1)
2012	8	7	-	1	-	-	1 (12.5)
2013	62	56	3	3	-	-	6 (9.7)
**Total**	317	285	6	21	5	-	32 (10.1)

**Note**: The test sera were screened at the initial dilution of 1:20.

### WB assays for antibodies to rM1 and rHA proteins

The 32 HI antibody positive sera were further explored by WB assay using rM1 or rHA protein derived from H1N1pdm virus as the test antigen. Due to lack of anti-elephant Ig-conjugate as the secondary detector, protein A/G conjugate was verified to replace this reagent.

**Fig 2:**
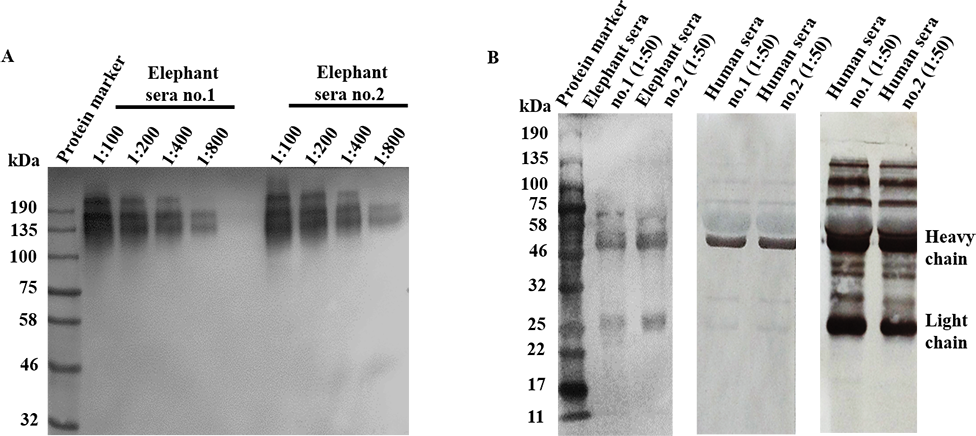
Binding efficiency of protein A/G-HRP to elephant serum Ig. (A) Under non-reducing conditions: protein A/G conjugate binds elephant IgG molecule at the MW of 150 kDa; (B) Under reducing conditions: protein A/G conjugate binds both H chain and L chain of elephant IgG with MW of 50 and 25 kDa, respectively (left panel). These MWs are identical to those of human IgG which reacts with HRP conjugated-goat anti-human IgG, IgM, IgA (H+L) and shows various bands of proteins with different MWs, suggesting of H chains and L chains of different Ig classes (right panel). The result also demonstrated that H chain, but not L chain of human Ig, binds protein A/G efficiently (middle panel).

The diluted elephant sera were allowed to bind protein A/G conjugate under the non-reducing or reducing condition. Under the non-reducing condition, a single protein band with MW of 150 kDa was visualized. This MW corresponded to the IgG molecule of elephants as previously reported by other groups of investigators [26–28]. The results obtained from 2 elephant serum samples were similar (Fig 2A). The WB assay conducted under the reducing condition demonstrated 2 bands of elephant serum proteins with MWs of 50 and 25 kDa, corresponding to the H and L chains of IgG, respectively as compared to human Ig (Fig 2B). It was concluded that protein A/G conjugate could bind the elephant Ig efficiently.

### WB assay for antibody to rM1 and rHA proteins in elephant sera

**Fig 3:**
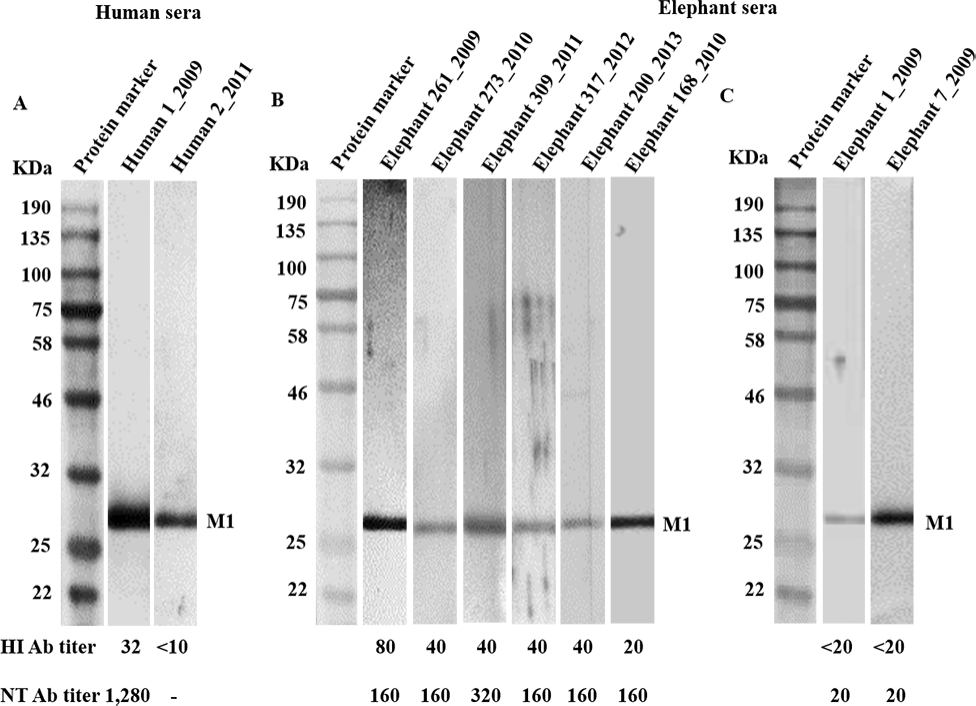
Detection for antibody to H1N1pdm M1 using rM1 protein with MW of 27 kDa as the test antigen in WB assay. (A) Two human serum samples: A convalescent serum of a patient infected with H1N1pdm virus with HI titer 320 and a serum sample from a blood donor with HI titer <10 using rabbit anti-human IgG-HRP as the secondary antibody; (B) Elephant sera with different HI titers; and (C) Elephant sera with HI titers <20. Protein A/G conjugate is used as the secondary detector for elephant Ig.

All 32 HI positive and 5 HI negative serum samples were determined for presence of antibody to H1N1pdm M1 and HA proteins by WB assay using recombinant proteins derived from A/California/04/2009 (H1N1) virus as the test antigen. Moreover, 10 serum samples from blood donors with HI titers to H1N1pdm virus in the range between <10 and 160 (6 cases with titer of <10, 1 case with titer of 20, 2 cases with titer of 40, and 1 case with titer of 160) were included for comparison. The result showed that all 37 elephants and all 10 blood donors tested were positive for antibody to rM1 antigen (Fig 3). It is likely that the elephants and blood donors with HI negative antibody might have been previously infected with other influenza virus strains.

**Fig 4:**
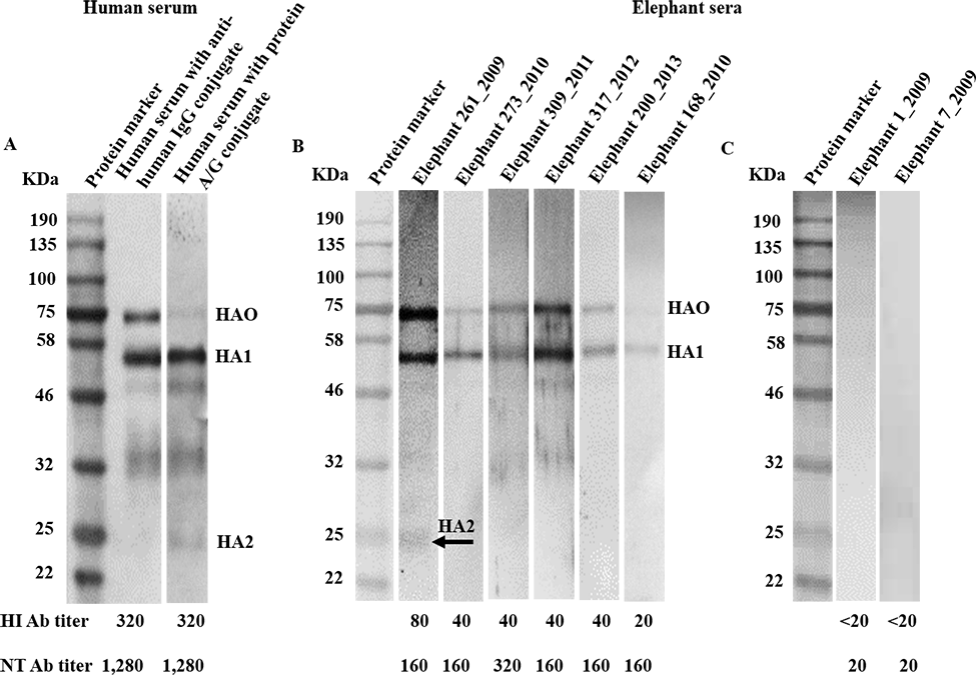
Detection of antibody to H1N1pdm HA using rHA protein as the test antigen in WB assay. (A) Convalescent serum from an H1N1pdm patient recognized the HA0, HA1 and HA2 proteins with MW of 75, 55 and 25 kDa, respectively as using anti-human Ig conjugate or protein A/G conjugate as the secondary detector; (B) Elephant sera with HI antibody titers ≥20 recognized HA0 and/or HA1 proteins, only one serum sample also recognized the HA2 domain; and (C) Elephant sera with HI antibody titer <20 does not react with H1N1pdm HA antigen. Protein A/G conjugate is used as the secondary detector for elephant sera.

A convalescent serum sample from an H1N1pdm patient with HI antibody titer of 320 was used as the reference control in assay for antibody to rHA antigen. This serum sample recognized 3 bands of rHA proteins with MWs of 75, 55 and 25 kDa, corresponding to the HA0, HA1 and HA2 domains, respectively (Fig 4A). The study of elephant sera showed that 27 of 32 HI antibody-positive samples were positive for HA antibody, comprising 2 of 6 samples with HI titer 20, and 25 of 26 samples with HI titers ≥40. Of all 27 positive samples, only 1 serum sample with HI titer of 80 recognized all 3 HA domains: HA0, HA1 and HA2; 21 with HI titers of 40 and 80 recognized 2 HA domains: HA0 and HA1; and 5 serum samples with HI antibody titers of 20 and 40 recognized only HA1 domain (Fig 4B). As negative serum controls, all 5 elephant serum samples with HI antibody titer <20 did not recognize any HA protein domain (Fig 4C).

#### Summarization on detection of H1N1pdm antibody using various methods

**Fig 5:**
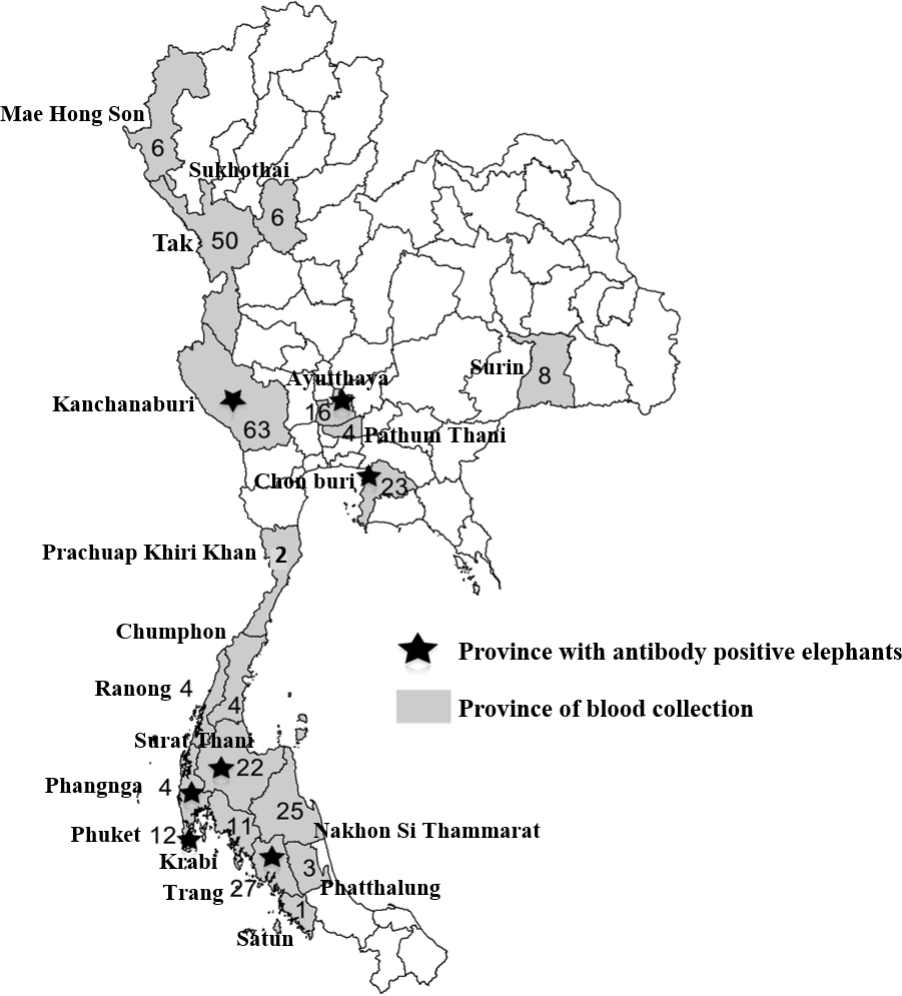
Geographical distribution of domestic elephants infected with H1N1pdm virus in Thailand.

In general, HI assay is used to identify the strain of influenza virus isolate and detect strain-specific antibody [29]. In this study, HI assay was used as the screening test for H1N1pdm antibody, and the seropositive rate of 10% (32 of 317) was obtained (Table 1). These HI positive sera were further verified by microNT assay for neutralizing antibody and WB assays for antibodies to M1 and HA protein. The result showed that microNT assay and WB assay for M1 antibody could not differentiate between HI positive and HI negative sera. On the other hand, WB assay for HA antibody yielded the positive result with HI positive sera only; and the positive rate of 8.5% (27 of 317) was obtained. Therefore, the overall seropositive rate of 8.5% was conclusive for the elephant sera studied (Table 2). With an analysis from results displayed in Tables 1, 2 and 3, the prevalence of H1N1pdm antibody by years were 2% (4/172) in 2009, 32% (17/53) in 2010, 9% (2/22) in 2011, 12% (1/8) in 2012, and 5% (3/62) in 2013. The elephants in younger age-groups were higher in seropositive rate than those from the older age-groups (Table 2). Interestingly, these H1N1pdm positive sera were collected from elephants living in tourist cities of Thailand: Ayutthaya, Chon Buri, Kanchanaburi, Phangnga, Phuket, Surat Thani and Trang (Fig 5 and Tables 2 and 3).

**Table 2:** Prevalence of H1N1pdm antibody in elephant sera by age-group, year and location.

Age-group	Number of sera screened by HI	Number of positive sera at screening by HI	Number of confirmed positive sera (%)	Year (number and location of positive samples)
1–10	24	3	3 (12.5)	2010 (2 samples from Phuket)
				2013 (1 sample from Kanchanaburi)
11–20	41	5	4 (9.8)	2010 (3 samples from Phuket)
				2013 (1 sample from Kanchanaburi)
21–30	42	4	2 (4.8)	2009 (1 sample from Surat Thani)
				2010 (1 sample from Phuket)
31–40	56	1	1 (1.8)	2009 (1 sample from Trang)
41–50	28	2	2 (7.1)	2010 (1 sample from Phuket)
				2013 (1 sample from Kanchanaburi)
51–60	11	1	1 (9.1)	2009 (1 sample from Phangnga)
>60	10	0	0 (0)	0
Unknown age	105	16	14 (13.3)	2009 (1 sample from Kanchanaburi)
				2010 (4 samples from Ayutthaya, 1 sample*)
				2010 (5 samples from Phuket)
				2011 (2 samples from Chon Buri)
				2012 (1 sample from Chon Buri)
**Total**	317	32	27 (8.5)**	

* denotes unknown location

**5 serum samples with HI antibody titers of 20 (4 samples) and 40 (1 sample) are excluded.

**Table 3:** Prevalence of H1N1pdm antibody in elephant sera by province and year.

Province	Year of blood collection	Number of tested sera	Number of positive sera (%)
Ayutthaya	2010	16	4 (25)
Chon Buri	2011	16	2 (12.5)
	2012	7	1 (14.3)
Chumphon	2009	4	0 (0)
Kanchanaburi	2009	1	1 (100)
	2013	62	3 (4.8)
Krabi	2009	11	0 (0)
Mae Hong Son	2009	6	0 (0)
Nakhon Si Thammarat	2009	25	0 (0)
Pathum Thani	2009	4	0 (0)
Phangnga	2009	4	1 (25)
Phatthalung	2009	3	0 (0)
Phuket	2010	12	12 (100)
Prachuap Khiri Khan	2010	2	0 (0)
Ranong	2009	4	0 (0)
Satun	2009	1	0 (0)
Sukhothai	2009	6	0 (0)
Surat Thani	2009	12	1 (8.3)
	2010	10	0 (0)
Surin	2009	1	0 (0)
	2011	6	0 (0)
	2012	1	0 (0)
Tak	2009	50	0 (0)
Trang	2009	27	1 (3.7)
Unknown	2009	13	0 (0)
	2010	13	1 (7.7)
**Total**		317	27 (8.5)

## Discussion

After causing the influenza pandemic in 2009, the H1N1pdm virus has been transmitted to several animal species as demonstrated by virus isolation, genome detection and serological assays [5, 8–17]. This serosurveillance study, which demonstrated the presence of H1N1pdm antibody in elephants, indirectly showed that this human influenza strain has crossed species barriers to infect elephants. The serological assays in animals frequently encountered the problem of non-specific serum inhibitors that could yield false positive results or enhanced antibody titers in HI and microNT assays [25, 30–35]. Another obstacle is the lack of anti-species Ig-conjugate needed as the secondary detector in enzyme-linked immunosorbent assay (ELISA) or WB assay. Our WB assay has overcome this problem by using the protein A/G conjugate as previously reported for several microbial agents in several animal species [36–43]. Our result demonstrated that protein A/G conjugate bound elephant IgG through the Fc-region of both H and L chains.

Non-specific inhibitors against influenza viruses in native sera of various animal species have been classified into three types: α-, β-, and γ-inhibitors based on their thermal stability, virus-neutralizing activity, and sensitivity to inactivation by the sialidase treatments [34, 44]. The amount of serum inhibitors found in various animal species may vary [35]. This study used both RDE and kaolin followed by heat inactivation for removal of non-specific serum inhibitors. RDE inactivates the α and γ-inhibitors [45–47], kaolin removes the γ-inhibitors [48, 49] and heating at 56°C inactivates the β-inhibitors [50–52]. However, no treatment method was perfect to remove all kinds of non-specific inhibitors in human and animal sera [32, 35]; and no single treatment method was found to be effective against all virus strains tested [35]. To our experience, the treatment method employing RDE and kaolin combination yielded clearer HI patterns when tilting the reaction plate for reading the antibody titers.

Of 317 elephant sera investigated, 32 were positive for H1N1pdm antibody by HI assay. Based on reasons mentioned above, these positive numbers might include the true positive result against the H1N1pdm virus, cross-reactive antibody against heterologous influenza A strains, and false positive result from the leftover of non-specific inhibitors. Therefore, the HI positive sera together with the HI negative serum controls were further investigated by microNT assay and WB assays for H1N1pdm M1 and HA proteins. The result showed that both microNT assay and WB assay for the rM1 protein could not differentiate between HI positive and HI negative sera; while WB assay for HA antibody was reactive with HI positive sera only. Moreover, all 6 human serum samples with HI titer <10 were also positive for rM1 antibody. It is well documented that NT antibody directs against both HA1 and HA2 domain, and HA2 domain is a conserved protein across influenza A strains. On the other hand, HI antibody reacts only with the HA1 domain, therefore it is more strain specific. [25, 53–54]. A monoclonal antibody directed against the HA2 domain of HA molecule could neutralize heterologous influenza A virus subtypes [55]. Moreover, our previous work found that the native sera from naïve laboratory guinea pigs could neutralize various influenza A subtypes, probably due to the action of non-specific serum inhibitors [30]. Similar to the HA2 domain, M1 is also a conserved protein across influenza A subtypes [56]. Our analysis found that M1 was the most conserved protein across all 18 HA subtypes of influenza A viruses based on clustalW multiple alignment (BioEdit Sequence Alignment Editor version 7.2.5.0), and 4 of 5 human serum samples collected during the pre-pandemic period of H1N1pdm virus (between 2004 and 2008) contained antibody to H1N1pdm M1 protein as determined by WB assay (unpublished data). Therefore, the positive H1N1pdm M1 antibody might be due to the cross-reactive antibody induced by the other influenza A strains. It is likely that influenza A virus infections have gone unrecognized in elephant population for a long time. According to broad activity of the antibody detected, our study suggested that microNT and WB assay for M1 antibody are not suitable for detection of antibody against specific strain of influenza A viruses. On the other hand, our data suggested that WB assay for HA antibody was more specific than microNT and WB assay for M1 antibody.

Collectively, the prevalence of H1N1pdm antibody in elephants decreased from 10 to 8.5% (range 2.3 to 32.1%) when HI assay was used in adjunct with WB assay for HA antibody. These elephants were at ages ranging from 5 to 52 years, and the H1N1pdm viral infection was more common in young elephants. By the time of infection, the prevalence of H1N1pdm antibody gradually increased from 1.5% in the first half of 2009 to 2.9% in the second half of the same year. Interestingly, all of the antibody-positive elephants came from the tourist cities of Thailand. For example, a positive rate of 100% was obtained from 12 elephants living in Phuket, a famous seaside province, and from 25% (4 of 16) of elephants in Ayutthaya, a UNESCO World Heritage old capital of Thailand.

The source of H1N1pdm infection in elephants is unknown, but most likely came from mahouts who got the viral infection from tourists or directly from the infected tourists who engaged in activities such as elephant riding or feeding. Moreover, it cannot be excluded that the infection might occur through elephant-to-elephant transmission. The prevalence of antibody in these elephants was in line with that found in the human population of Thailand. Epidemiological and virological studies reported that Thailand was first attacked by the H1N1pdm virus in May-June 2009 with peak of the first epidemic wave between July and August 2009 [57, 58]. It was estimated that the infection rate of H1N1pdm virus among blood donors in Bangkok in September 2009 was about 7%, and it was about 3% in the general adult population in Chiang Mai and Nakhon Sri Thammarat in December 2009 [25].

The number of animal species that come into close contact with humans is increasing, partly as the result of human behaviors such as tourism and exotic pet animals. Human transmission of H1N1pdm virus to a variety of animal species have been demonstrated by several groups of investigators. It is not known whether this is due to an increased interest in exploring potential sources of the next pandemic virus, or this virus strain has higher transmissibility than the others. Nevertheless, the greater the number of susceptible animal species, the higher the rates of genetic variation and risk of pandemicity.
